# A rodent version of the Iowa Gambling Task: 7 years of progress

**DOI:** 10.3389/fpsyg.2014.00203

**Published:** 2014-03-18

**Authors:** Ruud van den Bos, Susanne Koot, Leonie de Visser

**Affiliations:** ^1^Department of Organismal Animal Physiology, Faculty of Science, Radboud University NijmegenNijmegen, Netherlands; ^2^Division Behavioural Neuroscience, Department of Animals in Science and Society, Faculty of Veterinary MedicineUtrecht University, Utrecht, Netherlands; ^3^Department of Neuroscience and Pharmacology, Brain Centre Rudolf Magnus, University Medical Centre UtrechtUtrecht, Netherlands

**Keywords:** decision-making, humans, rats, prefrontal cortex, foraging behavior, behavioral models

## Abstract

In the Iowa Gambling Task (IGT) subjects need to find a way to earn money in a context of variable wins and losses, conflicting short-term and long-term pay-off, and uncertainty of outcomes. In 2006, we published the first rodent version of the IGT (r-IGT; Behavior Research Methods 38, 470–478). Here, we discuss emerging ideas on the involvement of different prefrontal-striatal networks in task-progression in the r-IGT, as revealed by our studies thus far. The emotional system, encompassing, among others, the orbitofrontal cortex, infralimbic cortex and nucleus accumbens (shell and core area), may be involved in assessing and anticipating the value of different options in the early stages of the task, i.e., as animals explore and learn task contingencies. The cognitive control system, encompassing, among others, the prelimbic cortex and dorsomedial striatum, may be involved in instrumental goal-directed behavior in later stages of the task, i.e., as behavior toward long-term options is strengthened (reinforced) and behavior toward long-term poor options is weakened (punished). In addition, we suggest two directions for future research: (1) the role of the internal state of the subject in decision-making, and (2) studying differences in task-related costs. Overall, our studies have contributed to understanding the interaction between the emotional system and cognitive control system as crucial to navigating human and non-human animals alike through a world of variable wins and losses, conflicting short-term and long-term pay-offs, and uncertainty of outcomes.

## INTRODUCTION

In 1994, Bechara and colleagues published the first paper on the Iowa Gambling Task (IGT; Bechara et al., 1994). In this task subjects need to find a way to earn money in a context of variable wins and losses, conflicting short-term and long-term pay-off, and uncertainty of outcomes. The IGT mimics daily, real-life, decisions (Damasio, 1994) and has given a strong impetus to understanding the role of the emotional system in the organization of decision-making behavior as well as the role of different prefrontal structures herein (e.g., Bechara, 2005; de Visser et al., 2011a; Gläscher et al., 2012). Furthermore, it has proven to be a useful neuropsychological tool to assess deficits in decision-making behavior underlying disorders related to, e.g., anxiety, eating, and addiction (reviews: Dunn et al., 2006; van den Bos and de Ridder, 2006; de Visser et al., 2011a; van den Bos et al., 2013a).

A number of rodent versions of the IGT (r-IGT) have been published during the last decade (van den Bos et al., 2006a; Pais-Vieira et al., 2007; Rivalan et al., 2009; Zeeb et al., 2009), allowing studying general, cross-species, principles underlying decision-making at a behavioral and a neural level (review: de Visser et al., 2011a). Elsewhere, we have reviewed IGT-like decision-making behavior related to eating behavior (van den Bos and de Ridder, 2006), different r-IGT models (de Visser et al., 2011a), neural structures (de Visser et al., 2011a), sex differences (van den Bos et al., 2013b), social modulation (van den Bos et al., 2013c), stress (van den Bos et al., 2013c) and (pathological) gambling (van den Bos et al., 2013a). Here, we review emerging ideas on the involvement of the emotional system and cognitive control system in r-IGT task-progression, i.e., we discuss the involvement of different prefrontal-striatal networks underlying task-progression (see IGT: Involvement of Prefrontal Structures). In addition, we suggest two directions for future research: (1) the role of the internal state of the subject in decision-making, and (2) studying differences in task-related costs (see New Directions for the r-IGT). We start by introducing our r-IGT (see A Rodent Model of the IGT) and end with a few general remarks (see Final Remarks ).

## A RODENT MODEL OF THE IGT

In 2001 Spruijt, van den Bos, and Pijlman published a review in which they discussed, among others, the economy of animal behavior: which neurobiological mechanisms underlie foraging-related decision-making behavior in animals such that long-term behavior is, by and large, optimal (Spruijt et al., 2001). As discussed by Cabanac (1971, 1992) emotions are important causal factors in steering behavior toward the best long-term option (Cabanac, 1971: *pleasant is useful*). Similar ideas have emerged from studies using the IGT (Damasio, 1994; Bechara et al., 1997, 1999). We therefore adopted the IGT as research-tool to address questions related to guiding behavior toward a long-term optimal solution and underlying neural circuits (van den Bos, 2004; van den Bos et al., 2006a).

To model the IGT we developed a choice-box with one arm containing 1 sugar pellet with 2 out 10 times a quinine-saturated sugar pellet (8 pellets win per 10 choices; “long-term advantageous arm”) and one arm containing 3 sugar pellets with 9 out of 10 times quinine-saturated sugar pellets (3 pellets win per 10 choices; “long-term disadvantageous arm”; van den Bos et al., 2006a; de Visser et al., 2011a). Thus, in this way we introduced a conflict between short-term and long-term pay-off of options as in the human IGT (de Visser et al., 2011a). We also introduced two empty arms as a control for non-specific effects, such as related to memory. Recently, we have automated the task for use in the home-cage (Koot et al., 2009a, 2012; de Visser et al., 2011a).

When we compare the performance of rats and mice in the r-IGT to performance of humans in the IGT we observe similar patterns. In the first part of the task subjects explore the different options [first 40–60 trials in humans (100 trials in total), first 40–60 trials in animals (120 trials in total)], while in the second part they choose the long-term advantageous option more often (see van den Bos et al., 2006a). In contrast to other r-IGT models (see Rivalan et al., 2009; Zeeb et al., 2009) and the human IGT (Bechara et al., 1994) we have not differentiated between long-term outcome and frequency of reward/punishments of options in our r-IGT. However, given the strong similarity between our human and animal data thus far (e.g., de Visser et al., 2010, 2011b; van den Bos et al., 2012, 2013b), this has as yet not proven to be a setback or inherent problem of our task.

## IGT: INVOLVEMENT OF PREFRONTAL STRUCTURES

**FIGURE 1:**
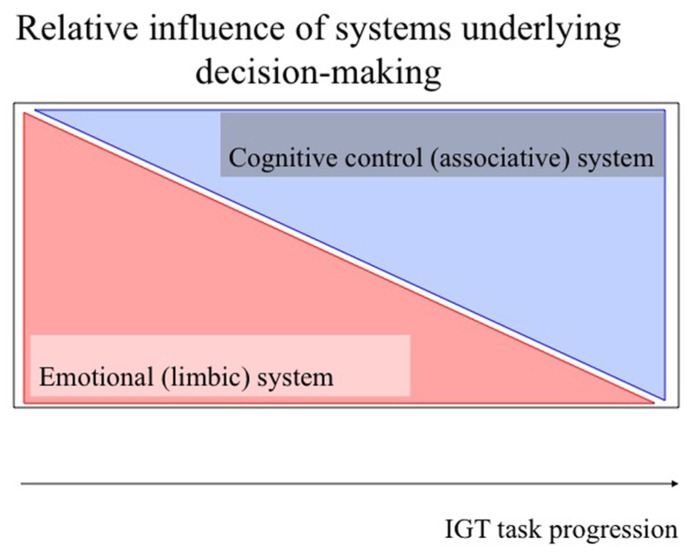
**Schematic model of the role of different systems in task-progression in the IGT.** The horizontal axis represents the progression of the task, while the vertical axis represents relative activity. The upper and lower triangle represent the relative contribution of the different brain systems which may be involved in the different stages of the test: learning relevant task-relevant features (emotional (limbic) system; transparent red), and consistently directing behavior toward choosing cards from the long-term advantageous decks [cognitive control (associative) system; transparent blue, see text for further explanation].

The output of decision-making processes, i.e., which action is taken in the end, is suggested to be determined by an interaction of two different forebrain systems: an emotional (limbic) system and a cognitive control (associative) system (e.g., McClure et al., 2004; Bechara, 2005; van den Bos et al., 2006b; de Visser et al., 2011a; Gläscher et al., 2012; **Figure 1**). During IGT performance these systems are activated in parallel, i.e., act as feed-forward and feedback systems, to optimize long-term behavior, and only differ in relative weight in different phases of the task (de Visser et al., 2011a). While the emotional system may be dominating the early phase in healthy individuals, the cognitive control system may be dominating the late phase, suppressing (eventually) activity in the emotional system.

At the level of prefrontal structures, in humans the emotional system may encompass the orbitofrontal cortex (OFC) and the ventromedial prefrontal cortex (VMPFC), while the cognitive control system encompasses the dorsolateral prefrontal cortex (DLPFC) and anterior cingulate cortex (ACC; e.g., McClure et al., 2004; Northoff et al., 2006; Lin et al., 2008; Lawrence et al., 2009; Li et al., 2010; de Visser et al., 2011a; Gläscher et al., 2012). The development of rodent versions of the IGT has led to the question whether activity of similar structures underlies IGT-like decision-making in rodents. This would not only enhance the validity of the models, but also allows for specific manipulations in different structures.

In our studies thus far, we clearly observed a role for the lateral orbitofrontal cortex and medial prefrontal cortex [infralimbic (IL) and prelimbic (PrL) cortex] in task-performance (de Visser et al., 2011b,c; van Hasselt et al., 2012; Koot et al., 2013, 2014). More specifically, focussing on the medial prefrontal cortex, we observed that inactivation of the PrL cortex was effective when rats already chose for the long-term advantageous option, but not when they were still exploring the different options (de Visser et al., 2011c). In contrast, manipulations with the IL cortex were effective, regardless of whether rats were still exploring or already chose for the long-term advantageous option (Koot et al., 2014). Thus, these data tend to suggest that activity in the IL cortex may precede activity in the PrL cortex. If so, one would predict that a correlation will be found between c-Fos expression (as marker of neuronal activity; see de Visser et al., 2011b; van Hasselt et al., 2012; Koot et al., 2013) in the IL cortex and task-performance in trial block 51–60 when only 60 trials are given (conform de Visser et al., 2011b; van Hasselt et al., 2012; Koot et al., 2013), while no such correlation will be found for the PrL cortex. Pilot experiments have confirmed this prediction. Given that data from different experiments seem to converge to the notion that the IL cortex may be (functionally) equivalent to the VMPFC in humans, while the PrL cortex may be equivalent to the dACC and DLPFC in humans (Milad and Quirk, 2012; Gass and Chandler, 2013; Mihindou et al., 2013), data in the r-IGT seem to match the data in the human IGT (conform de Visser et al., 2011a). These findings are in line with data which suggest that the IL and PrL may play different roles in the organization of behavior, such as shown in studies in fear-conditioning (Milad and Quirk, 2012), appetitive behavior (Burgos-Robles et al., 2013; Horst and Laubach, 2013), and control in addictive behavior (Gass and Chandler, 2013; Mihindou et al., 2013).

**FIGURE 2:**
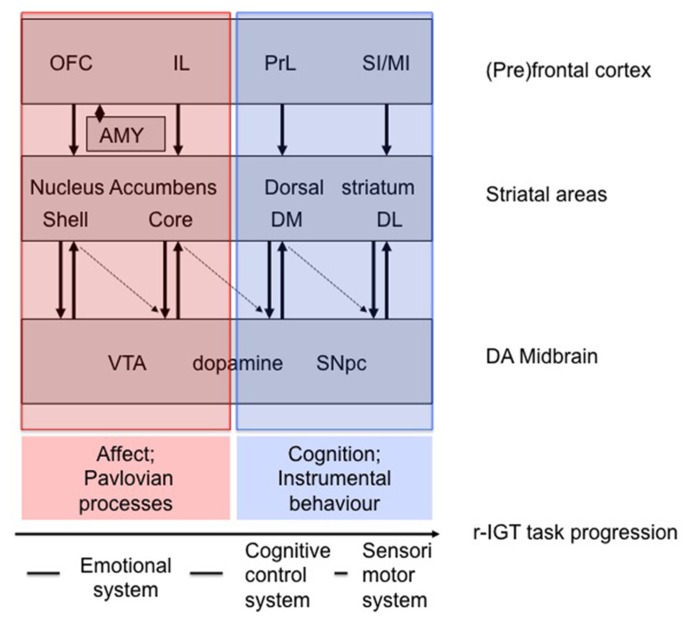
**Hypothetical neurobehavioral model of task-progression in the r-IGT (Yin and Knowlton, 2006; Yin et al., 2008).** It should be noted that the cingulate areas and insular cortex are not included (see New Directions for the r-IGT). Furthermore the subdivisions of the OFC are not shown (see van den Bos et al., 2013b). In transparent red, the emotional system is shown, of which striatal areas are involved in Pavlovian behavior (see Yin et al., 2008): while the shell is involved in immediate (hedonic) responses (stimulus-outcome; (un)conditioned consummatory/hedonic responses), the core is involved in anticipatory/preparatory behavior (stimulus–stimulus relation). In transparent blue, the cognitive control system [dorsomedial (DM) striatum; action-outcome; goal-directed behavior] and sensorimotor/habit system [dorsolateral (DL) striatum; stimulus-response; habit-like behavior], of which striatal areas are involved in instrumental behavior. Thus far, we have not trained animals to the point of showing habitual behavior. Arrows indicate mutual interactions between midbrain dopaminergic areas and striatal areas, while the dotted arrows indicate disinhibition of dopaminergic areas by striatal areas [see Yin and Knowlton (2006) for discussion]. Dopaminergic projections to the prefrontal cortex are not shown. Also the interaction with the serotonergic (5-HT) system is not shown (see for discussion: Homberg et al., 2008; Koot et al., 2012; van den Bos et al., 2013b). Abbreviations: amy, amygdala; OFC, orbitofrontal cortex; IL, infralimbic cortex; PrL, prelimbic cortex; SI/MI, primary and sensory motor cortices; VTA, ventral tegmental area; SNPc, substantia nigra pars compacta.

In general, our findings on the involvement of prefrontal areas in r-IGT performance are in line with those of other studies (Rivalan et al., 2011; Zeeb and Winstanley, 2011; Paine et al., 2013; Pittaras et al., 2013; Zeeb and Winstanley, 2013). Next to r-IGT related performance differences in activity in prefrontal areas we have observed task-related performance differences in activity in striatal areas (e.g., de Visser et al., 2011b). **Figure 2** incorporates our findings in a broader perspective of prefrontal-striatal areas underlying r-IGT task-progression. This tentative neurobehavioral model of task-progression in the r-IGT is based upon models of cortico-basal ganglia systems (Yin and Knowlton, 2006; Yin et al., 2008). As discussed by Yin et al. (2008) areas encompassing the nucleus accumbens/ventral striatum are involved in Pavlovian processes, while areas encompassing the dorsal striatum are involved in instrumental behavior. When we more specifically relate this difference to the earlier discussion on the medial prefrontal cortex this amounts to the following tentative picture (see legend **Figure 2** for other areas). The core area of the accumbens has been implicated in anticipatory/preparatory behavior related to Pavlovian cues signaling the expected value of commodities (Yin et al., 2008). In similar vein, the VMPFC in humans is involved in anticipatory (Pavlovian) signaling of good *versus* bad options in the IGT aiding in directing decision-making behavior toward the best long-term option (Bechara et al., 1999), which has been framed in a broader context as “affective meaning” (Roy et al., 2012). Given the suggestion that the IL cortex in rats may be related to the VMPFC in humans (Milad and Quirk, 2012; Gass and Chandler, 2013; Mihindou et al., 2013), the IL cortex and core area of the nucleus accumbens in tandem may play a role in aiding to direct behavior toward the best long-term option by anticipating expected values of options. In contrast, the dorsomedial striatum is involved in organizing instrumental goal-directed behavior, i.e., in reinforcing behavioral acts and/or behavioral patterns which are conducive to reaching the goal, while punishing behavioral acts and/or patterns which deviate from reaching the goal (Yin et al., 2008; Paton and Louie, 2012; Kravitz et al., 2012). The PrL cortex as rodent equivalent of the dorsal ACC and DLPFC (Milad and Quirk, 2012; Gass and Chandler, 2013; Mihindou et al., 2013) may play a role in assessing final cost-benefit options of instrumental behavior by error-monitoring as well as outcome feedback, working memory and organizing goal-directed behavioral actions (Killcross and Coutureau, 2003; Ostlund and Balleine, 2005; Kolling et al., 2012). In tandem, therefore, the PrL and dorsomedial area of the striatum may play a role in organizing instrumental goal-directed behavior toward the best long-term option (Ostlund and Balleine, 2005).

In sum, these systems exert different levels of control over decision-making behavior (see van den Bos et al., 2006b; de Visser et al., 2011a; van den Bos et al., 2013b). The emotional system is involved in immediate responding to (potential) rewards, losses or threats (i.e., impulsive behavior) as well as in emotional control, i.e., adjusting behavior to changing contingencies and anticipating the value of intended choices. In this way it allows the organism to label the environment in terms of “long-term hot and not spots.” This emotional-laden information is input for the cognitive control system, which subsequently “organizes” instrumental behavior toward the best long-term option, i.e., this system is more involved in response inhibition, error-monitoring, switching and long-term/future perspectives. At a behavioral level this would amount to the differentiation between responses to emotional-laden stimuli, such as anticipatory responses, and developing consistent behavior toward the best-long-term option (instrumental learning).

Both the human IGT and our rodent version of the IGT are associative learning tasks which tap-off learning-related processes under conditions of uncertainty without any prior training, i.e., in the very early stages of processing information, and subsequently organizing a consistent behavioral response toward the best long-term option. Therefore, it is critical to assess to what extent neural findings underlying task-performance relate to other paradigms, which use more extensive training protocols. Thus, animals may have acquired competing responses during earlier training affecting subsequent behavior and activation of structures (Rivalan et al., 2011; see New Directions for the r-IGT).

## NEW DIRECTIONS FOR THE r-IGT

The r-IGT has contributed to understanding neurobiological mechanisms of how subjects may arrive at the best long-term option. Thus far, we have not systematically investigated the role of hunger levels on decision-making behavior. In our experiments we have used a very moderate level of food deprivation (90–95% of free feeding weight). However, increasing levels of deprivation may lead to different behavioral outcomes. It is known that hunger levels (or current energy budget) have an effect on decision-making, exploration, impulsivity and risk-taking behavior (Krebs and Davies, 1993; Inglis et al., 1997, 2001; de Visser, 2003; Koot et al., 2009b; Proctor, 2012). Thus, in the r-IGT both hunger levels before the task and increasing satiation during the task may have an effect on subsequent choices made. For instance, as subjects are extremely hungry they may become more risk-taking and focus on short-term rather than long-term options. The insular cortex may play a role in shifting between these behavioral strategies. For, this structure has been implicated in interoceptive awareness, homeostatic control and energy expenditure (Butti and Hof, 2010; Prévost et al., 2010; Craig, 2011). Furthermore, the insular cortex has connections with the dorsal and ventral striatum and thereby may exert an effect on immediate and long-term focus (see Tanaka et al., 2004). Moreover, we have already seen a relation between insular cortex activity and r-IGT performance in rats (van Hasselt et al., 2012; Koot et al., 2013), in line with other studies that have shown a relation between insular cortex activity and decision-making/risk-taking in rats and humans (Clark et al., 2008; Xue et al., 2010; Ishii et al., 2012). Thus, one direction for future research using the r-IGT may be to study the role of the internal state and insular cortex activity in decision-making.

In the r-IGT new information is acquired which is not integrated with earlier obtained information. However, in real “rodent” life, decision-making is an ongoing process of using earlier acquired information, checking/updating “known” options, and deciding to explore new options should they occur. More precisely, real-life decision-making exists of coding the value of options, assessing the overall value of the environment (rich/poor) and assessing whether to engage with a current option or move to another location (see Kolling et al., 2012). Studies in humans and animals have shown that the ACC may be critically involved in assessing levels of energy expenditure of instrumental behavior or actions in relation to reward, i.e., in assessing physical or action-related costs (Walton et al., 2003; Rudebeck et al., 2006; Croxson et al., 2009; Prévost et al., 2010; Cowen et al., 2012; Kolling et al., 2012). These studies have in addition shown that the OFC and VMPFC are more involved in delays and probabilities related to reward and punishments. In line with this both we (probabilities; e.g., de Visser et al., 2011b; van Hasselt et al., 2012) and Rivalan et al. (2011; delays) have seen only little correlation of c-Fos expression in cingulate areas with task-performance or effects of lesions of cingulate areas on task performance. Thus, costs may be dissected into different components with different underlying neural structures: physical (or foraging) costs related to instrumental behavior/actions associated with activity in the ACC, and costs associated with delays to reward and frequencies of punishments/omissions associated with activity in the OFC and VMPFC. From this perspective the barrier-climbing based decision-making task that we have used earlier (van den Bos et al., 2006c) may be remodeled to assess the effects of physical costs on IGT-like performance. In addition, new tasks may be developed. For instance, in which animals have learned the value of different options in an environment (costs associated with frequencies/delays), and subsequently are presented a choice between a pair of options with a relatively low pay-off (but one slightly better than the other) and a pair of options with a relatively high pay-off (but one slightly better than the other) associated with a physical (or foraging) cost, for instance, by climbing barriers; or, for instance, a choice between a known pair and a completely novel option associated with a foraging cost. Recently, such paradigms in humans have dissected the role of the ACC (engage or leave; foraging decision) and VMPFC (decision based on differences within a pair of options) in decision-making behavior (Kolling et al., 2012). Thus, a direction for future research using the r-IGT may be to study the role of foraging decisions, foraging costs and ACC activity in decision-making.

## FINAL REMARKS

Here we discussed a few aspects related to IGT-like decision-making, i.e., decision-making in a context of variable wins and losses, conflicting short-term and long-term pay-offs, and uncertainty of outcomes. Our interest for engaging into the IGT was fuelled by questions related to understanding mechanism underlying long-term successful foraging behavior in animals (Spruijt et al., 2001; van den Bos, 2004). The r-IGT that we developed has contributed to understanding the interaction between the emotional system and cognitive control system as crucial systems in this respect. Recently, we have discussed how to bridge the gap between these mechanisms and evolutionary models that focus on the function or long-term consequences of behavior (van den Bos et al., 2013c). Along with understanding the role of the internal state and understanding different task-related costs, this will be one of the challenges for future research.

## Conflict of Interest Statement

The authors declare that the research was conducted in the absence of any commercial or financial relationships that could be construed as a potential conflict of interest.
